# Personality Traits and Cardiotoxicity Arising From Cancer Treatments: An Hypothesized Relationship

**DOI:** 10.3389/fpsyg.2021.546636

**Published:** 2021-05-05

**Authors:** Ilaria Durosini, Ketti Mazzocco, Stefano Triberti, Gaetano Alessandro Russo, Gabriella Pravettoni

**Affiliations:** ^1^Applied Research Division for Cognitive and Psychological Science, IEO, European Institute of Oncology IRCCS, Milan, Italy; ^2^Department of Oncology and Hemato-Oncology, University of Milan, Milan, Italy; ^3^Independent Researcher, Milan, Italy

**Keywords:** cardiotoxicity, cancer, cardiovascular disease, patients, cancer treatments

## Abstract

Thanks to the evolution in medical and pharmaceutical research, to date, the number of cancer treatments is increasingly on the rise. Despite this, several side effects related to cancer treatments can exacerbate patients’ physical and psychological conditions, such as cardiotoxicity. Over the years, researchers have explored the possible relationship between psychological variables and physical diseases. Even though some authors examined the relationship between personality and specific diseases, no scientific attention has been paid to the role of personality in the development of cardiotoxicity arising from cancer treatments. Yet this is an important objective, given that determining whether personality influences cardiac toxicity of anticancer treatments could inform the processes by which stable psychological factors influence health. This contribution summarizes and analyzes the available scientific evidence about the association between personality and main cardiotoxicity-related-diseases of anticancer therapies, including cancer and cardiovascular diseases, in order to sketch a hypothetical model of the relationship between personality traits and cardiotoxicity.

## Introduction

The imperative nature of cancer mortality captures the attention of healthcare professionals, patients, and citizens globally. In the last decades, the number of therapeutic cancer treatments has rapidly increased (e.g., Smith et al., 2010), but adverse treatment-related toxicity remains a relevant problem (Floyd et al., 2005). Many anticancer drugs and targeted therapies have direct or indirect detrimental effects on the cardiovascular system, worsening patients’ quality of life, limiting the possibility of further therapy, and increasing the mortality rate (Hewitt et al., 2003; Senkus and Jassem, 2011; Han et al., 2017). Also the American College of Cardiology/American Heart Association (ACC/AHA; Bonow et al., 2005) guidelines recognize the relevance of collateral effects, according to which patients who received chemotherapy are considered Stage A heart failure subjects with an increased risk for cardiac dysfunction. One of the most clinically relevant cardiac collateral effects is cardiotoxicity arising from cancer treatments (Senkus and Jassem, 2011; Curigliano et al., 2016; Trapani et al., 2020). This condition is defined as one of the most devastating sequelae of treatments that lead to heart failure, hypertension, left ventricular dysfunction (Bowles et al., 2012; Chen et al., 2012), cardiomyopathy, and coronary artery diseases (Taunk et al., 2015). The European Association of Cardiovascular Imaging and American Society of Echocardiography defined cardiac toxicity as a decline of LVEF greater than 10% points, with a final LVEF <53% (Plana et al., 2014). The onset of cardiotoxicity can occur at different times and is classified by some researchers as *acute cardiotoxicity* (immediately during treatment), *early progressive cardiotoxicity* (within the 1st year after completion of therapy), and *late progressive cardiotoxicity* (at least 1 year after completion of therapy; Wouters et al., 2005; Yeh and Bickford, 2009).

Despite the empirical evidence regarding the greater importance of psychological, environmental, and social aspects in healthcare diseases, cardiotoxicity is frequently associated with biomedical and drugs factors only, and the psychological perspective is often overlooked. However, humans go beyond the subcellular level and more attention should be given to the psychological dimensions related to health diseases (Fioretti et al., 2016). Studies showed that psychological factors may impact cancer progression and development and collateral effects occurrence (Spiegel and Kato, 1996; Butow et al., 2000; Turner-Cobb et al., 2001; Segerstrom, 2003), as well as patient engagement and commitment to treatment (Kondylakis et al., 2019; Griffin et al., 2020).

A psychological aspect that has often been explored in relation to health is personality traits (Hampson, 2012). The associations between personality and health hold constant across decades (Hampson et al., 2007; Johansen, 2018) and are related to health biomarkers (Hampson et al., 2013), physician-rated health (Chapman et al., 2007), and longevity (Roberts et al., 2007; Kern and Friedman, 2008; Jokela et al., 2013). Researchers have also found a relationship between personality traits and health-related behaviors, such as smoking involvement (Malouff et al., 2006; Munafo et al., 2007), alcohol consumption (Malouff et al., 2007; Hakulinen et al., 2015), vegetable/fruit intake (Lunn et al., 2014), and physical activity (Rhodes and Smith, 2006; Wilson and Dishman, 2015); also, the traumatic experience of cancer demonstrated to influence patients’ identity and personality (Triberti et al., 2019). Over the years, the possible links between personality and health has been largely defined by several models (Cohen, 1979; Contrada et al., 1990; Wiebe and Smith, 1997). For example, the *Stress Moderation Model* maintains that human traits may represent biological differences that contribute to disease. This model posits that stress causes illness and dispositional factors make people more or less vulnerable to its effects. The *Constitutional Predisposition Model* maintains that the associations between personality traits and disease may be correlational rather than causal. This model assumes that people may be genetically predisposed to certain pathophysiological processes which influence the development of illness and the cognitive, emotional, and behavioral aspects of personality. The *Health Behavior Model* proposes that personality affects health *via* the quality of one’s health practices. Personality is linked to illness because it affects people’s tendency to engage in unhealthy or healthy behaviors (e.g., smoking and lack of physical exercise).

Even though some authors examined the relationship between personality and health, no scientific attention has been paid to the role of personality in the development of cardiotoxicity arising from cancer treatments. However, determining whether personality influences cardiac toxicity of anticancer treatments could inform the processes by which stable psychological factors influence health, and provide valuable information for precision medicine. Taking into consideration the *Stress Moderation Model* and the *Health Behavior Model*, we will summarize and analyze the available scientific evidence about the association between personality and main cardiotoxicity-related-diseases, including cancer and cardiovascular diseases, to sketch an hypothesis about the relationship between personality traits and cardiotoxicity. According to the models referenced above, the present article maintains that human dispositional characteristics may represent biological differences that can influence the sensitivity to cardiotoxicity arising from cancer treatments; also, we will explain how personality affects behavior especially in terms of emotion regulation and health management, with an indirect effect on illness and disease.

## Personality, Cancer, and Cardiovascular Diseases

The first step toward a hypothesis about the role of personality in cardiotoxicity requires to analyze the literature on psychological traits and related diseases. Although conflicting results exist in the literature, some studies have shown that some peculiar human psychological aspects can emerge in relation to some health diseases. Some of these aspects are related to human personality and the related management of emotions, in particular. Indeed, the emotional management is a human ability (or a “life skill”; Martin and Ochsner, 2016; Mazzocco et al., 2019) that could be trained and empowered (Sebri et al., 2020), yet is heavily influenced by stable dispositions. For example, positive psychological characteristics such as optimism are correlated with good self-rated health (Smith et al., 2004) and are a predictor of positive physical health outcomes (Rasmussen et al., 2009). Thus, emotions are important social signals and, generally, people may show a tendency to express or withdraw them. According to personality, some psychological processes may operate on a conscious level (for example, conscientious persons would be more at unease in front of messy contexts or violation of rules), while others may impact at an unconscious level (for example, neurotic people will be more likely to feel and be more vulnerable to negative affect), leading to (a) inhibition or (b) excessive expression of negative emotions.

### Personality Characterized by Inhibition of Negative Emotions

Studies revealed that personality traits characterized by the tendency to inhibit negative emotions or the impairment in identifying and expressing inner feelings (i.e., alexithymia) may play a major role in health management. Some of the personality traits included in this category are Type-C personality (Temoshok, 1987) and “distressed”/Type-D personality (Denollet et al., 1996). The missed or limited expression of emotions and the difficulty in identifying feelings and distinguishing between emotions may generate detrimental consequences for one’s well-being (Kennedy-Moore and Watson, 2001) and higher risk of mortality for cancers and heart diseases (Grossarth-Maticek et al., 1985, 1988; Taylor et al., 1997; McCann, 2020).

In cancer research, these results were supported by Hürny and Adler (1981), who suggested that cancer patients can be characterized by some tendencies, including denial and reduced discharge of emotions, impaired expression of anger, self-sacrifice, and self-accusation, flat and vulnerable interpersonal relationships, as well as inhibited sexuality. Basically, the listed clusters share the tendency to inhibit and repress basic impulses, feelings, or emotions. In 1993, Scherg (1993) conducted a cross-sectional and longitudinal study with 2.340 women without evidence of cancer at the onset of the study and identified several premorbid psychological factors associated with the diagnosis of breast cancer among which were the tendency to repress anger and ignore occurrences related to illness. These results were subsequently confirmed in a 10-year follow-up study in which authors found that helpless or stoic patients were more likely to relapse (Pettingale, 1984).

It is interesting to note that personality traits characterized by the tendency to inhibit negative emotions expression and a deficit in emotional awareness may be associated with the development of cardiovascular diseases and heart failure too. Denollet and colleagues found that denying emotions and not sharing them with others were the risk factors for heart diseases (Denollet, 1998, 2005; Denollet et al., 2000; Hirokawa et al., 2004; Husson et al., 2015). A cross-sectional study with 3.813 participants showed that personality traits characterized by the tendency to experience negative emotions, but to inhibit them and their expression in social interactions (i.e., Type-D personality) were associated with coronary heart disease and hypertension after adjustment for age and sex (Denollet, 2005). Additionally, the difficulty understanding, identifying, describing, and expressing feelings and emotions appear to hinder adaptive emotional regulation, making alexithymia a risk factor for cardiovascular diseases (Peters and Lumley, 2007). Waldstein et al. (2002) highlighted that alexithymia in older adults is associated with several psychosocial characteristics that may predispose to cardiovascular diseases. The authors found that alexithymic older adults show greater blood pressure reactivity to anger provocation compared to non-alexithymics adults. Enhanced cardiovascular reactivity during emotionally intense circumstances may thus represent a pathway by which alexithymia relates to poor cardiovascular health outcomes.

Therefore, it seems that human personality traits characterized by the tendency to suppress negative emotions (Bleiker et al., 2008), the perception of most stressors as more stressful (e.g., Cea et al., 2002; Molerio and García, 2004), and to put aside personal needs to satisfy another’s desires (Eskelinen and Ollonen, 2011) are linked to greater risk of cancer, cardiac diseases, and shorter survival of patients with already established diagnoses (e.g., Nakaya et al., 2006; Chida et al., 2008). For this reason, some decades ago, it emerged the idea of a “cancer-prone personality,” which was investigated at large before being disconfirmed by longitudinal studies. For example, Bleiker et al. (2008) conducted a 13-year follow-up study in which they assessed the relationship between personality factors and breast cancer, showing that personal traits were not statistically significantly associated with an increased risk of this kind of cancer.

However, if it is not scientifically correct to say that personality could be considered a concurrent cause of cancer, data show that individual predispositions may promote repetitive emotional, behavioral, and experiential patterns that influence patients’ ability to deal with symptoms and manage their own health (Johansen, 2018).

Possible mechanisms that mediate these relationship include the accumulated stress responses (Eysenck, 1994), that disrupt the immune and endocrine systems (Garssen and Goodkin, 1999; Antoni and Lutgendorf, 2007; Zhu et al., 2021), influence the course of neoplastic disease (Sklar and Anisman, 1981), increased inflammation (Hänsel et al., 2010), determine poor treatment adherence, and unhealthy lifestyle (Reich and Schatzberg, 2010).

### Personality Characterized by Excessive Expression of Negative Emotions

Other personality tendencies that have been associated with cancer and cardiac diseases are related to the *excessive expression* instead of inhibition of emotions. Studies highlighted that people with personality traits typically related to the expression of negative emotions (i.e., competitive and hostile emotions and behaviors) tend to show a greater risk of death from clinical illness (e.g., Friedman and Rosenman, 1959; Rosenman and Friedman, 1974; Weiss et al., 2020). In this line, Type A personality (competitive, achievement-oriented, and aggressive) has been associated with cardiovascular disease risk (Sirri et al., 2012), although reviews deemed evidence about cardiovascular death inconclusive (Šmigelskas et al., 2015). The assumption that emotionally-excessive personalities in general may be related to health risk was backed by findings in earlier studies by Kissen and Eysenck, which identified neuroticism (general emotional lability, emotional over-responsiveness, and liability to neurotic breakdown under stress) and extraversion (outgoing and uninhibited social disposition) as potential risk factors for cancer (Kissen and Eysenck, 1962; Eysenck, 1985).

The association between personality traits characterized by the tendency to express negative emotions and health diseases was confirmed by Chang et al. (2002), who found that human disposition to react to stressful situations with intense emotion of anger were strongly associated with premature cardiac diseases and myocardial infarction. These results were subsequently confirmed in Suls and Bunde (2005) review, in which anger, anxiety, and depression appeared to be related to increased cardiac risk in healthy samples.

## Personality and Cardiotoxicity of Anticancer Treatments: Is There a Relationship?

Despite the idea of a “cancer-prone personality” has been abandoned, we started this contribution with the observed relation between some aspects of human personality traits, cancer, and cardiovascular diseases. Just like in the development of cancer and cardiovascular disease, we expect personality traits to influence the evolution of cancer treatment and the related toxic side effects.

**Figure 1:**
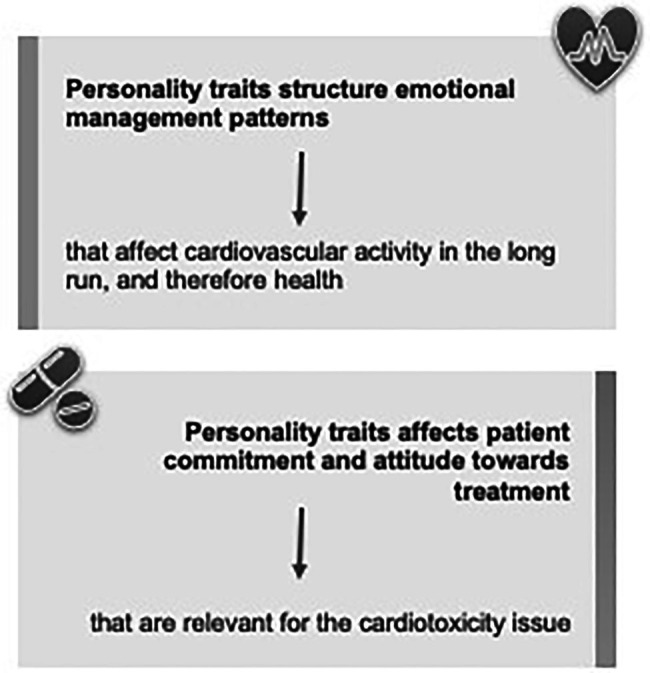
A schematic description of the role of personality traits in emotional regulation and health behaviors.

Personality traits work as important variables for emotional regulation and health behaviors:

1. First, personality traits structure emotional management patterns and habits (e.g., inhibiting emotions or extremely expressing them) that affect cardiovascular activity in the long run, and, therefore, health;
2. Second, individual traits influence one’s ability to manage symptoms and treatment (Figure 1).

For what regards the first modality, literature identifies some risk factors that could be associated with the risk of cardiotoxicity of anticancer therapies: among them is autonomic system dysregulation or the repeated activation (or extended stressful conditions) of the autonomic system (Musselman et al., 2000; Dong and Chen, 2018). Another risk factor for cardiotoxicity is high blood pressure (Panjrath and Jain, 2006; Sawaya et al., 2011; dos Santos et al., 2017), which is involved in emotional management, especially in terms of reduced emotional response to relevant stimuli (McCubbin et al., 2012, 2014). Accordingly, personality traits are associated with behavioral and psychophysiological patterns that could determine such factors, especially in terms of reduced or extreme emotional activation. In this sense, one’s psychological dispositions have a role in shaping persons’ autonomic system activation profiles, possibly resulting in risk factors for the response to cancer drugs. Besides, accumulated stress responses disrupt the endocrine and immune systems (Garssen and Goodkin, 1999; Antoni and Lutgendorf, 2007; Zhu et al., 2021), influence the course of neoplastic disease (Sklar and Anisman, 1981), and increase chronic inflammation (Hänsel et al., 2010).

For what regards the second modality, personality traits have important consequences in patients’ behavior related to health management. In this sense, personality is relevant for the cardiotoxicity issue because it affects patient commitment and attitudes towards treatments. Individual predispositions may promote repetitive emotional, behavioral, and experiential patterns that influence patients’ ability to deal with symptoms and cancer treatments. How a patient manages his emotions according to his personality characteristics could predispose patients to non-adequate and risky behaviors, such as smoking and substance consumption, as well as to conduct relevant for effective health management (e.g., Dahl, 2010). For example, neurotic and extroverted traits also appear to be associated with unhealthy behaviors and lifestyle (e.g., smoke: Spielberger and Jacobs, 1982; Thornton et al., 1994; Arai et al., 1997; alcohol intake: Cook et al., 1998; Pardo et al., 2007), exposing patients to the risk of incurring in serious health complications, especially when pertaining to drug therapeutics, thereby improving the risk of future cardiotoxicity events.

This risk also appears to be exacerbated by the influence of personality on patient adherence to treatment. Personality traits are of significant importance for adherence behavior (Axelsson et al., 2011; Hazrati-Meimaneh et al., 2020); people defined as “a worrying kind of person” (i.e., neuroticism; Costa and McCrae, 1992) tend to adhere low and in an inappropriate way to management suggestions (Bruce et al., 2010; Axelsson et al., 2011). On the contrary, conscientious people, because of their tendency to trust their abilities to manage their lives, tend to adhere to the prescribed treatment and abide to doctors’ advice (Rosengren et al., 2004; Pruette and Amaral, 2021), while extraverted and agreeable individuals are favored in developing positive relationships with health providers and caregivers, so to make use of social support facilitating health management (Berkman et al., 2000; Hazrati-Meimaneh et al., 2020).

## Discussion

Over the years, some researchers have explored the possible relationship between psychological variables and physical diseases. Even though some authors examined the relationship between personality and specific diseases, no scientific attention has been paid to the role of personality in the development of cardiotoxicity arising from cancer treatments. Yet this is an important objective, given that determining whether personality influences cardiac toxicity of anticancer treatments could inform the processes by which stable psychological factors influence health. This contribution summarizes and analyzes the available scientific evidence about the observed correlation between personality traits, cancer, and cardiovascular diseases in order to sketch a hypothetical model of the relationship between personality traits and cardiotoxicity. Indeed, part of the literature could led us to hypothesize that personality may be related to cardiotoxicity. Studies suggested that human traits are associated with the examined health diseases; personality dispositions work as important variables for emotional regulation, health behaviors, and management of stressful events. After a cancer diagnosis, patients are exposed to intense emotional responses, and they are called to handle this emotional burden. Personality traits are relevant precisely because of their role in managing emotions and the individual inner world. Mismanagement of emotions exposes cancer patients to a high emotional burden from having to handle them, and individual personality plays an important role here. How a patient manages his emotions according to his personality characteristics could also determine the course of treatment.

Just like in the development of cancer and cardiovascular disease, we expect personality traits to influence the efficacy of cancer treatment and the related toxic side effects. In conclusion, considering personality as a factor affecting the disease, the assessment of personality traits becomes crucial. In the light of a precision medicine approach, this will allow identifying treatment solutions centered on the individual, who should be profiled at a genomic but also at a psychological level. Further research is needed in order to produce stronger evidence on the role of personality traits in cardiotoxicity. In the last years, precision medicine and eHealth resources began to profile patients in order to strengthen individual health monitoring as well as the development of each patient’s healthcare journey. More precise profiling of patients, based on the main component of health (physical, psychological, and social), will be of help to health professionals in their decision-making process, allowing them to make the best decision based on patients’ unique information rather than on information based on previous experience with other patients (Mazzocco and Cherubini, 2010). In this sense, it is important to adopt a multidisciplinary and personalized standpoint, which would be necessary to manage all contributing factors. From the point of view of psychological assessment and diagnostic procedures, it is important to take into account personality characteristics, mental functioning, and symptom patterns. During assessment, people may adopt a defensive stance and/or lie when responding to tests, because of personality tendencies and/or potentially sensitive topics (Fantini et al., 2017). It may be necessary to adopt collaborative approaches that go beyond the sole consideration of symptoms taxonomies, becoming able to describe psychopathological pictures in terms of personality patterns, social, and emotional abilities, unique profiles as well as the subjective experience of illness, as prescribed, for example, by the literature on the Psychodynamic Diagnostic Manual (PDM-2; Lingiardi et al., 2015; Lingiardi and McWilliams, 2017; Hilsenroth et al., 2018). This approach guarantees a better information flow among health professionals (Gilardi et al., 2014), which in turn will improve the overall quality of care. The understanding of the structure and dynamics of personality must be a priority to assist with this kind of patient and potentially improve outcomes.

## Author Contributions

ID conceptualized the ideas presented in the article, wrote the first draft, and supervised revisions. KM and ST edited the manuscript and contributed to conceptualization and revision. GR conceptualized the ideas presented in the article and edited the first draft. GP contributed with important intellectual content and supervised the whole process. All authors contributed to the article and approved the submitted version.

### Conflict of Interest

The authors declare that the research was conducted in the absence of any commercial or financial relationships that could be construed as a potential conflict of interest.
